# Nit1 is upregulated in non-small cell lung cancer and promotes cancer cell proliferation and invasion and regulates EMT-related molecules and cyclins

**DOI:** 10.7150/jca.96095

**Published:** 2025-07-01

**Authors:** Jian Wang, Biying Jiang, Xiaojing Wang, Haifeng Liu, Chuifeng Fan

**Affiliations:** Department of Pathology, First Affiliated Hospital and College of Basic Medical Sciences of China Medical University, 110001, Shenyang, China.

**Keywords:** cyclin, EMT, invasion, Nit1, NSCLC, proliferation

## Abstract

Nitrilase homolog 1 (Nit1) is a member of the carbon-nitrogen hydrolase family whose function in human cancer is largely unknown. In this study we investigated the expression and function of Nit1 in non-small cell lung cancer (NSCLC) *in vivo* and vitro. Immunohistochemistry study shows that expression of Nit1 was elevated in non-small cell lung cancer compared to normal lung epithelial cells including submucosal glands and bronchial epithelial cells (p<0.05). Expression of Nit1 was significantly associated with advanced TNM stages, lymph node metastasis and poor clinical outcome of the patients (60.70 ±5.48 vs 30.83 ± 4.88) (p<0.05). Western blot also shows elevated expression of Nit1 in cancer tissues compared to adjacent normal lung tissues (p<0.05). We detected Nit1 expression using Western blot in lung cancer cell lines including A549, H460, H661, H1299, LK2, PC9, and SK, and bronchial epithelial cell line HBE. Immunofluorescent staining shows that Nit1 was located in cytoplasm in HBE, A549, H1299, H460 and PC9 cells. Overexpression of Nit1 in H1299 cells significantly promoted cancer cell proliferation and invasion *in vitro* (p<0.05). Western blot study confirmed that overexpression of Nit1 in H1299 cells significantly upregulated EMT-related molecules including MMP3, Slug, snail, and cyclins including cyclin D1, cyclin D3 and cyclin E, and downregulated EMT-related molecule E-cadherin. These results indicate that Nit1 expression was upregulated in non-small cell lung cancer and may contribute to the malignant phenotype through regulating EMT-related molecules and cyclins.

## Introduction

Nitrilase homolog 1 (Nit1) belongs to the carbon-nitrogen hydrolase family 1,2. Human Nit1 has 327 amino residues and a carbon-nitrogen hydrolase domain. It disposes of the deaminated glutathione by catalyzing the hydrolysis of the amide bond in N-(4-oxoglutarate)-L-cysteinylglycine 1,2. The function of nit1 in mammals was inspired by the study of the function of nitfhit protein in Drosophila melanogaster and Caenorhabditis elegans 3,4. Unlike mammalian Nit1 and Fhit proteins, which are encoded by different isolated genes, Nit1 and Fhit fuse into a protein which was encoded by the NitFhit gene in Drosophila melanogaster and Caenorhabditis elegans 4. Although some studies have shown that the loss of FHIT function is related to the acceleration of tumor cell proliferation, the role of FHIT in tumor is still controversial. Semba's study indicates that deletion of Nit1 gene can accelerate the proliferation of some tissue cells in mice 5. However, the interaction of Nit1 and FHIT and the biological effects in mammalian cells are still lack of experimental verification. The function of Nit1 in human cells including cancer cells is largely unknown. Wang *et al.* found that Nit 1 was overexpressed in lung cancer and downregulation of Nit1 decreased overall cell survival of lung cancer cells in culture 6. However, the significance of Nit1 in lung cancer tissues and the molecular mechanism of its role in lung cancer is unclear. However, the molecular mechanism involved in its function was not uncovered. In this study, we investigate the expression of Nit1 in human non-small cell lung cancer. We focus on the clinicopathological significance of Nit1 expression in cancer tissues *in vivo* and the related molecular regulation in cancer cells *in vitro*.

## Materials and methods

### Patients and tissue samples

Tissue samples included NSCLC tissues and paired adjacent lung tissues. The Patients received surgery in the First affiliated hospital of China Medical University, without any preoperative radiotherapy or chemotherapy. The patients do not have any major diseases that can affect their prognosis. Histological types of the tumors were confirmed according to the World Health Organization (WHO) classification of tumors of the lung, pleura, thymus, and heart 7 by at least two pathologists. There were 41 cases of squamous cell carcinomas (SCCs) and 52 cases of adenocarcinomas. The Institutional Review Board of China Medical University approved this study. All patients have given informed consent for this study.

### Immunohistochemistry

An SP-kit was used for the immunostaining assays according to the manufacturer's instructions. Formalin-fixed, paraffin-embedded tissue samples were sliced into sequential 4 μm-thick sections. After dewaxing in xylene and gradual rehydration in a graded ethanol series, the sections underwent heat-induced antigen retrieval in citrate buffer (pH 6.0) by autoclaving for 90 seconds (until flame shutoff), followed by an additional 30 seconds of cooling with cold water. To minimize background interference, endogenous peroxidase activity was quenched with 3% H2O2, and non-specific binding was blocked using non-immune sera. The sections were subsequently treated with Nit1 antibody (sc-515566, Santa Cruz, CA, USA, dilution, 1:50) overnight at 4°C. Protein detection was performed using a catalyzed signal amplification system (Maixin Biotechnology, Fuzhou, China) following the manufacturer's protocol. Signal visualization was achieved via the avidin-biotin complex (ABC) method, employing biotinylated secondary antibodies (Maixin) and ABC reagent (Maixin), with diaminobenzidine (DAB) as the chromogen. Finally, the sections were lightly counterstained with hematoxylin, dehydrated through an alcohol series, and mounted for analysis. The sections were evaluated by three independent observers, who were blinded to the patients' clinical data. In cases of disagreement, the investigators reviewed the findings together to reach a consensus. Immunohistochemical scoring was determined using two criteria: the percentage of immunopositive cells and their staining intensity. The proportion of positive cells was graded as: 0 (< 10%), 1 (≥ 10% to < 25%), 2 (≥ 25% to < 50%), 3 (≥ 50% to < 75%), and 4 (≥ 75%). Staining intensity was classified as: 0 (negative), 1 (weak), 2 (moderate), or 3 (strong). The final immunoreactivity score for each section was calculated by multiplying the two component scores. For statistical analysis, a score below 2 was classified as negative, while a score of 2 or higher was considered positive.

### Immunoblotting analysis

This assay was carried out and evaluated as described previously 9. The primary antibodies included: β-actin (sc-47778, Santa Cruz, CA, USA, dilution, 1:1000), Cyclin D1 (sc-8396, Santa Cruz, CA, USA, dilution, 1:500), Cyclin D3 (sc-6283, Santa Cruz, CA, USA, dilution, 1:500), Cyclin E1 (sc-377100, Santa Cruz, CA, USA, dilution, 1:500), Nit1 (sc-515566, Santa Cruz, CA, USA, dilution, 1:200), slug (sc-166476, Santa Cruz, CA, USA, dilution, 1:200), snail (#3879, Cell Signaling, MA, USA, dilution, 1:1000), MMP9 (sc-21736, Santa Cruz, CA, USA, dilution, 1:200), E-cadherin (sc-8426, Santa Cruz, CA, USA, dilution, 1:200), GAPDH (sc-47724, Santa Cruz, CA, USA, dilution, 1:500), and Myc-tag (sc-40, Santa Cruz, CA, USA, dilution, 1:500).

### Cell culture and transfection

The cell lines used in this study included HBE, LK2, H1299, A549, PC9, H460, H661 and SK. Cells were cultured according to the guideline of the ATCC/CTCC. Nit1 cDNA clone (Origene, RC211519, Rockville, MD, USA) was transfected using Lipofectamine 3000 (Invitrogen, Carlsbad, CA, USA) according to the manufacturer's instructions.

### Immunofluorescence

The detailed protocol was described previously 8. Briefly, cells were incubated with primary antibody for detecting Nit1 (sc-515566, Santa Cruz, CA, USA, dilution, 1:100). The secondary antibodies (1:200) were conjugated to rhodamine. Olympus IX51 fluorescent microscope (Olympus, Tokyo, Japan) was used for detecting immunofluorescent staining. Images were captured by CoolPIX 5400 camera (Nikon, Japan).

### MTT assay

The MTT assay was performed to assess cell viability and proliferation. Cells were seeded in 96-well plates at a density of 5×10³-1×10⁴ cells/well in complete medium containing 10% FBS and incubated for 24 hours to allow attachment. Subsequently, cells were treated with varying concentrations of the test compound or vehicle control (DMSO < 1%) for 48 hours. MTT reagent (5 mg/mL in PBS) was added to each well (10 μL/well), and plates were incubated at 37 °C for 4 hours to enable formazan crystal formation by metabolically active cells. The supernatant was aspirated, and formazan was dissolved in 100 μL DMSO per well with gentle agitation for 10 minutes. Absorbance at 550 nm (with 630 nm as a reference) was measured using a microplate reader. Data were normalized to untreated controls (set to 100% viability) and analyzed using GraphPad Prism. All experiments were conducted in triplicate, and error bars represent standard deviation. Controls included blank wells (medium only) and untreated wells to account for background signal and cell viability baseline, respectively.

### Colony formation

The detailed protocol was described previously 10. 1,000 cells were plated in 6 cm cell culture dishes and incubated for 10 days. Cells were treated with varying concentrations of the test compound or vehicle control (DMSO < 1%) for 48 hours. MTT reagent (5 mg/mL in PBS) was added to each well (10 μL/well), and plates were incubated at 37°C for 4 hours to enable formazan crystal formation by metabolically active cells. The supernatant was aspirated, and formazan was dissolved in 100 μL DMSO per well with gentle agitation for 10 minutes. Absorbance at 550 nm (with 630 nm as a reference) was measured using a microplate reader. Data were normalized to untreated controls (set to 100% viability) and analyzed using GraphPad Prism. All experiments were conducted in triplicate, and error bars represent standard deviation. Controls included blank wells (medium only) and untreated wells to account for background signal and cell viability baseline, respectively. The plates were stained with Giemsa, and colonies with more than 50 cells were counted under a stereomicroscope.

### Matrigel invasion assay

The detailed protocol was described previously 10. The Matrigel invasion assay was performed to evaluate cell invasive capacity. ​​24-well Transwell chambers​​ (Corning, 8 μm pore polycarbonate membrane) were pre-coated with ​​Matrigel​​ (BD Bioscience, 1 mg/mL) diluted in serum-free medium (1:8 ratio) and allowed to polymerize at 37°C for 4 hours. ​​Cell density optimization​​: Cells (5×10⁴ cells/well) in serum-free medium were seeded onto the Matrigel-coated upper chambers, while the lower chambers contained complete medium with 10% FBS as a chemoattractant. After 24-48 hours of incubation (cell type-dependent), non-invading cells were removed using cotton swabs. Invading cells on the membrane were fixed with ​​4% paraformaldehyde​​, stained with ​​0.1% crystal violet​​, and imaged under a phase-contrast microscope. Five random high-power fields (200×) per membrane were counted to quantify invasive cell numbers. All experiments were performed in triplicate, and data were analyzed using GraphPad Prism (mean ± SD).

### Statistical analysis

SPSS v22.0 (IBM, Chicago, IL, USA) was used for all the statistical analyses. Pearson's chi-square test was used for determination of clinicopathological significance of protein expression. Student's t-test was used for determination of differences between the groups. *P*-values < 0.05 were considered significant.

## Results

### NIT1 was upregulated in NSCLC and associated with cancer progression

**Figure 1:**
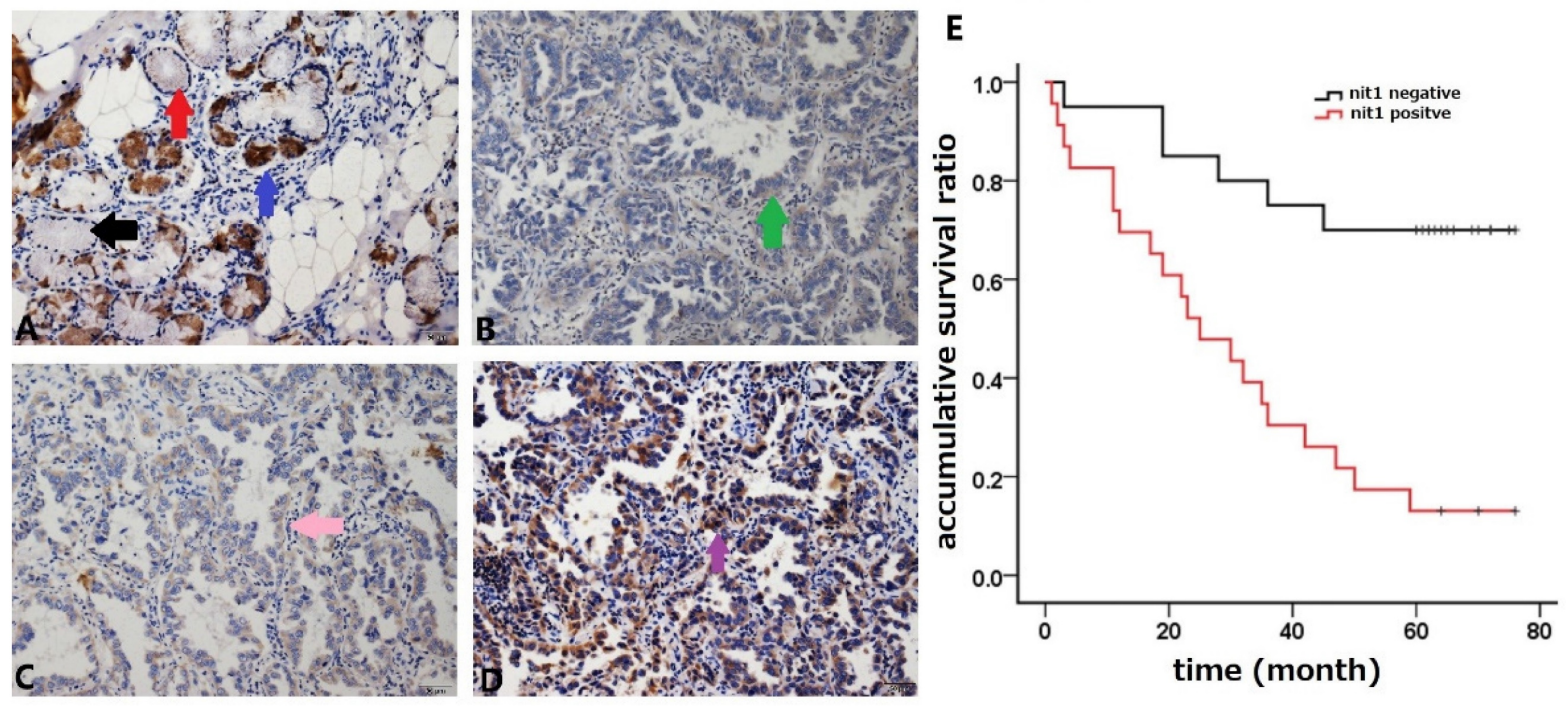
**NIT1 expression detected by immunostaining and analysis of the survival time.** Bronchial NIT1 immunostaining was mainly detected in cytoplasm including lung epithelial cells (A, the black arrow show no immunostaining of NIT1 in some epithelial cells, the red arrow shows weak cytoplasm immunostaining of NIT1 in some epithelial cells, and the blue arrow shows strong cytoplasm immunostaining of NIT1 in some epithelial cells) and cancer cells (B,C,D, the green arrow show weak cytoplasm immunostaining of NIT1 in cancer cells, the pink arrow shows median cytoplasm immunostaining of NIT1 in cancer cells, and the purple arrow shows strong cytoplasm immunostaining of NIT1 in cancer cells). The survival time of patients with NIT1 expression in cancer tissues was significantly shorter than that in without NIT1 expression (60.70 ±5.48 vs 30.83 ± 4.88) (p<0.05) (A: ×100; B, C, D: ×400)

**Figure 2:**
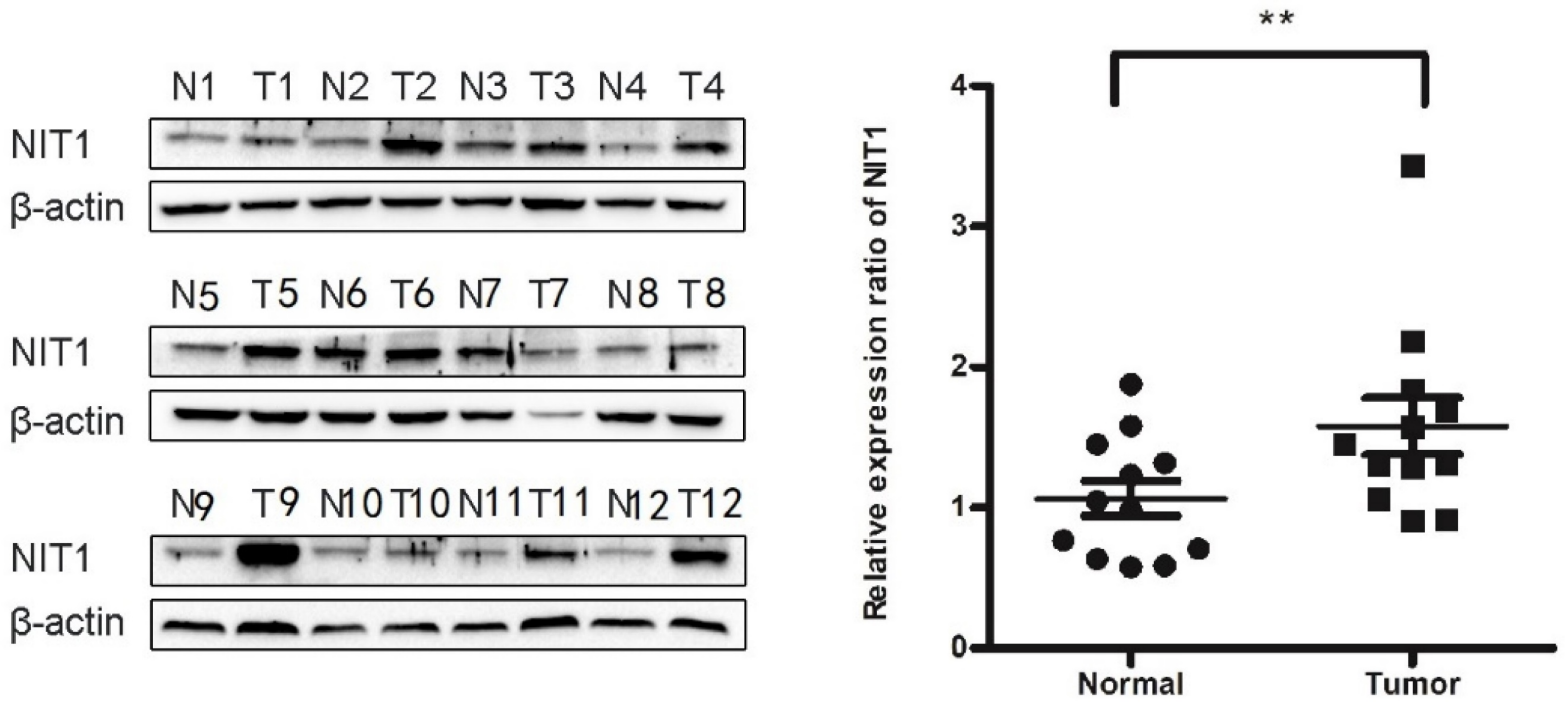
** Comparison of NIT1 expression in lung and cancer tissues using western blot.** NIT1 was detected in lung tissues and NSCLC tissues using Western blot. As the Scatter Plot shows NIT1 expression in NSCLC tissues was significantly higher than that in lung tissues (**p<0.05).

We used immunohistochemistry staining to detect NIT1 expression in normal lung tissues and NSCLC tissues. NIT1 expression was mainly detected in cytoplasm of normal lung epithelial cells and cancer cells (Figure 1). The positive rate of NIT1 expression in cancer tissues (64.5%, 60/93) was significantly higher than that in normal lung epithelial cells (17.1%, 6/35) (*p* < 0.05). NIT1 expression in NSCLC cells was significantly associated with poor differentiation of cancer cells, advanced TNM stages (III versus I+II), lymph node metastasis and poor clinical outcome of the patients ((60.70 ±5.48 vs 30.83 ± 4.88 months) (Table 1 and Figure 1) (p < 0.05). The positive rate of NIT1 in squamous cell carcinoma and adenocarcinoma was 65.9% (27/47) and 63.5% (33/52) respectively and shows no significant difference (p > 0.05). We also detected NIT1 expression in lung and cancer tissues using Western blot which showed that NIT1 was increased in cancer tissues compared to adjacent lung tissues (Figure 2, *p* < 0.05).

### NIT1 promotes lung cancer cell proliferation and invasion

**Figure 3:**
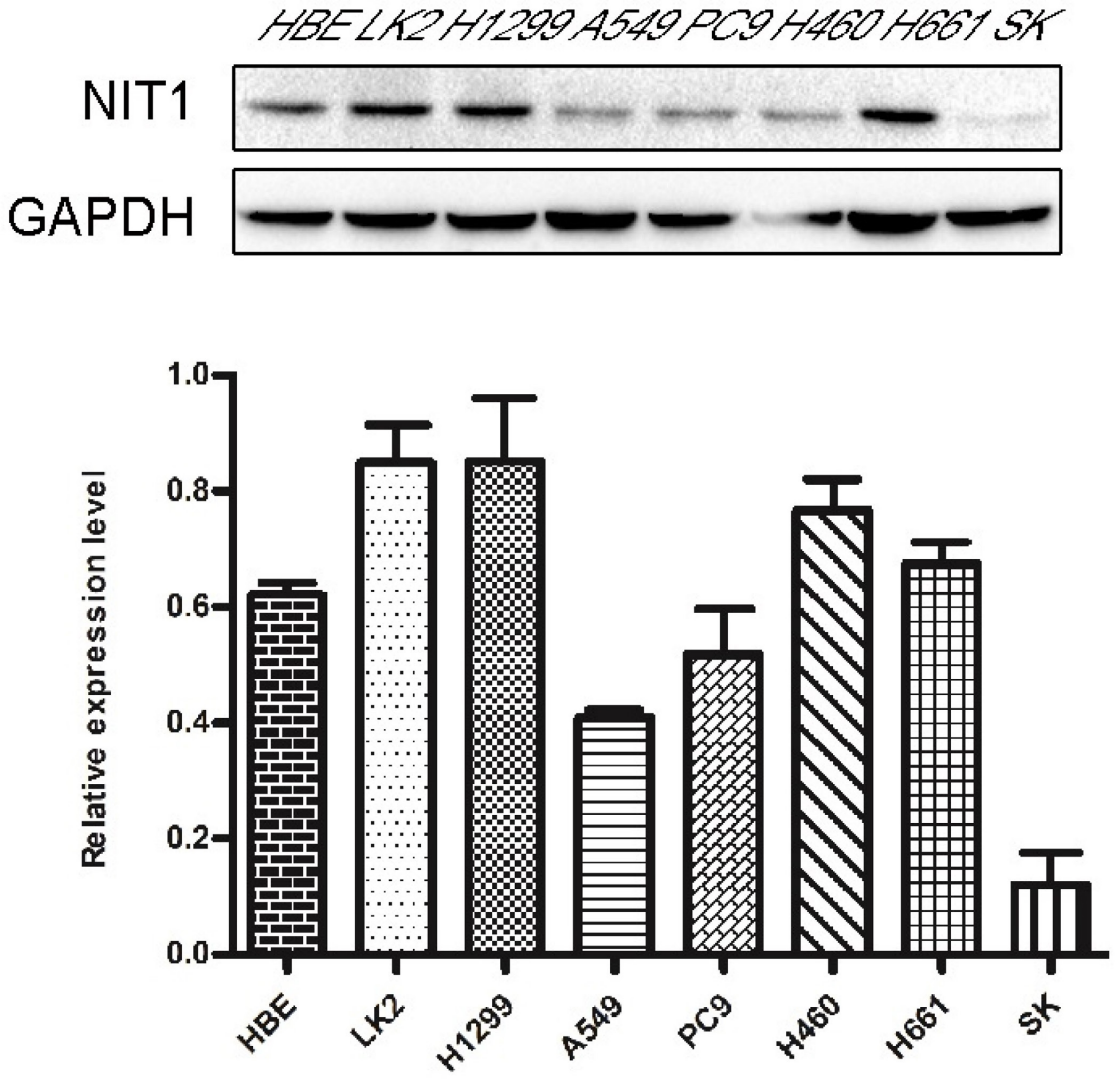
** Detection of NIT1 in cell lines *in vitro*.** Protein lysates from the indicated cell lines (HBE, LK2, H1299, A549, PC9, H460, H661 SK) were analyzed by Western blot to detect NIT1 (upper band) and GAPDH (lower band, loading control). Membranes were probed with specific antibodies, and protein bands were visualized using chemiluminescent detection. Gray-scale images are shown. A variable level of NIT1 expression was detected using Western blot in lung epithelial HBE cells and cancer cells including LK2, H1299, A549, PC9, H460, H661 and SK.

**Figure 5:**
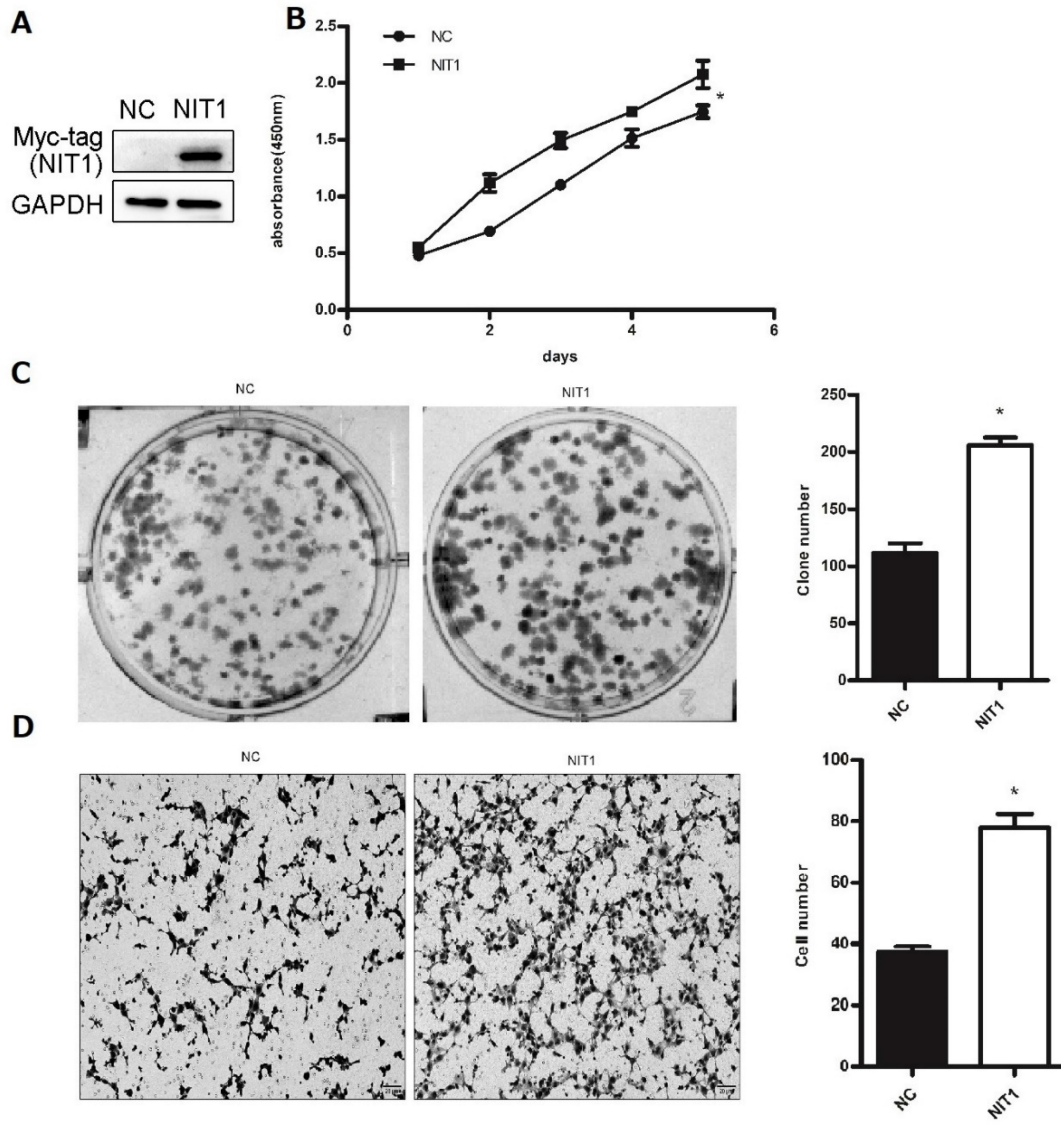
**NIT1 regulated cancer cell proliferation and invasion *in vitro*.** NIT1 was overexpressed using transfection of NIT1 cDNA clones (A). MTT (B) and colony formation study (C) showed that overexpression of NIT1 significantly promoted cancer cell proliferation (*p*<0.05). Transwell study showed that overexpression of NIT1 significantly promoted cancer cell invasion (D) (*p*<0.05).

We used Western blot and immunofluorescent staining to detect NIT1 expression in cell lines including lung epithelial HBE cells and cancer cells including A549, H1299, LK2, SK, PC9, H460 and H661 cells *in vitro*. Variable levels of NIT1 expression were detected in these cells (p < 0.05). Immunofluorescent staining showed that NIT1 expression in HBE, A549, H1299, H460 and PC9 cells was located in cytoplasm (Figure 3). Overexpression of NIT1 using transfection of NIT cDNA clones in H1299 cells significantly promoted cancer cells proliferation (MTT and colony formation studies) (Figure 5 A, B and C) and invasion (Transwell study) (Figure 5, D).

### NIT1 regulates EMT-related molecules and cyclins in lung cancer cells

**Figure 6:**
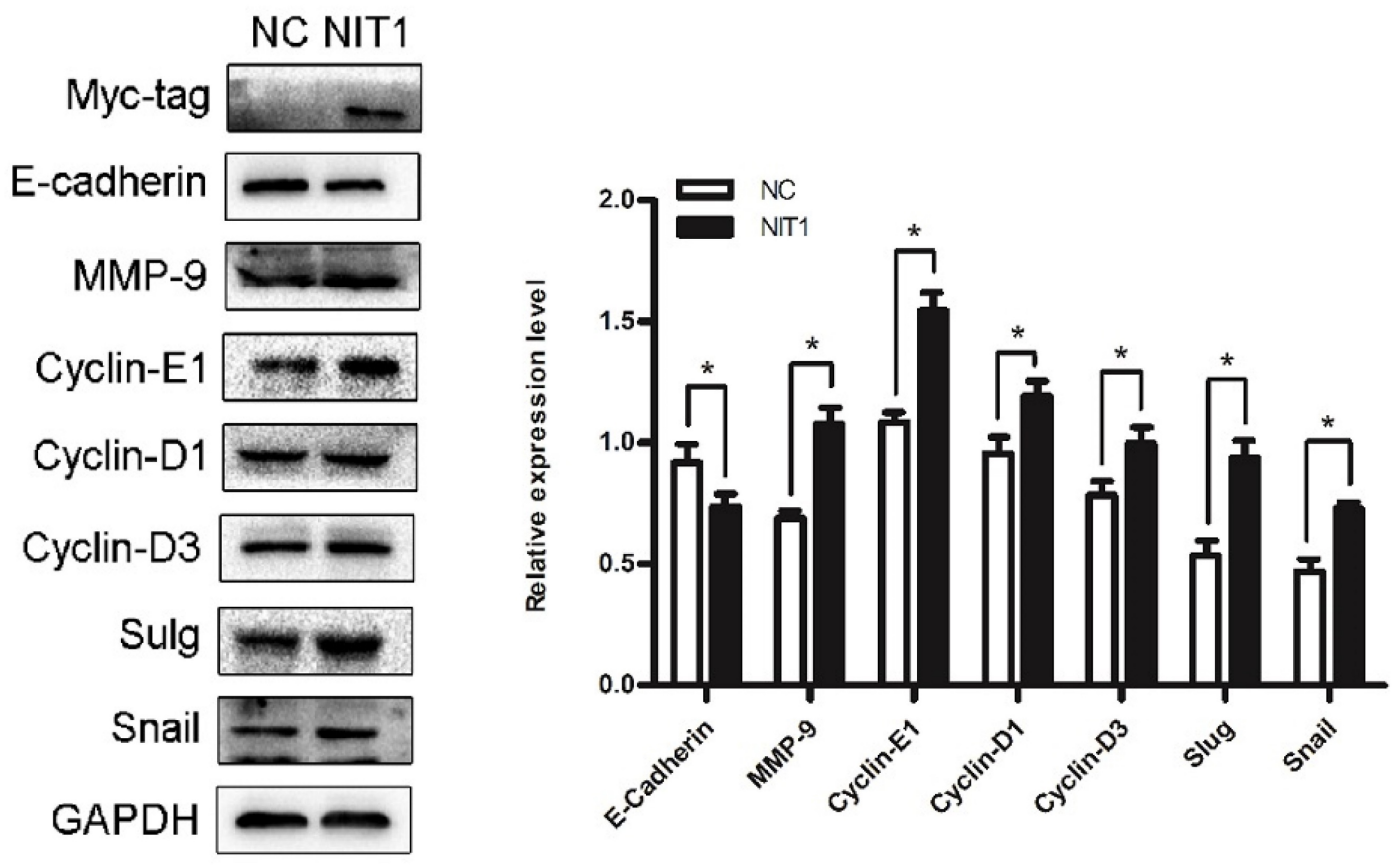
**NIT1 regulated EMT-related molecules and cyclins in H1299 cells.** Overexpression of NIT1 in H1299 cells significantly upregulated MMP9, cyclin D1, cyclin D3, cyclin E1, snail, and slug and downregulated E-cadherin expression (*p*<0.05).

We overexpressed NIT1 in H1299 cells using transfection of cDNA clones of NIT1 and using western blot to investigate the molecular regulation by NIT1. Western blot study showed that overexpression of NIT1 in H1299 cells significantly regulated EMT-related molecules and cyclins which are associated cancer cell proliferation and invasion. In detail, NIT1 significantly upregulated MMP9, slug, snail, cyclin D1, cyclin D3, and cyclin E1, and downregulated E-cadherin expression in H1299 cells (Figure 6, *p* < 0.05).

## Discussion

Peracchi *et al.* demonstrated that Nit1 is an amidase and its enzyme activity is different from that of NIT2, which has 35% sequence identical to Nit1 1. This study proved that Nit1 has activity toward deaminated glutathione. However, the function of Nit1 is largely unknown. At present, the role of nit1 in tumor is mainly based on the research in mice 3. Semba's study shows that Nit1 deficiency can accelerate the proliferation of renal cells, skin epithelial cells and mammary epithelial cells, and induce gastric tumor 3. This study suggests that nit1 may form a fusion gene with FHIT to regulate cell proliferation and tumorigenesis. However, the function of Nit1 in mammalian cells is largely unknown. Zhang's study proved that Nit1 has a physiological regulating function of T cells in mammalian cells 11. The studies of expression of nit1 in human tumors are still relatively rare. Sun's study demonstrated that Nit1 expression was detected in about half of the cases of esophageal adenocarcinomas 4. Wang *et al.* found that Nit1 was overexpressed in both lung cancer cells in culture and lung tissues 6, But the clinical significance of Nit1 in human lung cancer tissues and the possible molecular mechanism was not fully understood. In the current study, we found that the rate of Nit1 expression in NSCLC was 50% and the expression level of Nit1 was significantly higher than that in normal lung epithelial cells. We examined Nit1 expression in lung tissues and lung cancer tissues using both immunohistochemistry and western blot and both studies proved a higher level of Nit1 expression in cancer tissues compared to normal tissues. We also used Western blot and immunofluorescent staining to detect Nit1 expression in cells including bronchial epithelial cell HBE and lung cancer cells *in vitro*. The immunohistochemistry study in cancer cells and immunofluorescent staining both indicate that Nit1 expression was mainly found in cytoplasm. Furthermore, our study shows that overexpression of Nit1 significantly associated with TNM stages and survival time of the patients, which indicates that Nit1 may contribute to the progression of NSCLC. In Lin's study, Nit1 expression in colorectal cancer cells was significantly reduced compared to adjacent normal epithelial cells 12. The study shows that Nit1 contribute to the progression of colorectal cancer through regulating TGFβ1-Smad2/3 signaling pathway. In our study, Nit1 expression was found to be significantly associated with lung cancer progression which indicates Nit1 may have different function in different tumors. Our study further proved that Nit1 may promote lung cancer cell proliferation and invasion through regulating epithelial-to-mesenchymal transition (EMT) related molecules and cyclins. EMT is an important mechanism for cancer cells to maintain invasiveness 13. E-cadherin is an important adhesion molecule to maintain the junctions between epithelial cells. Reduced expression or dysfunction of E-cadherin is an important marker of EMT 5. The important mechanism of E-cadherin expression reduction is transcriptional inhibition. Slug and snail2 are two important transcription factors regulating the expression of E-cadherin 14. The abnormal expression of slug and snail is found in many malignant tumors including NSCLC 14,15. Cyclins are key factors in regulating cell proliferation 16. Our study confirmed that Nit1 regulates EMT-related molecules and cyclins, which may explain its function in regulating cancer cell proliferation and invasion. However, the molecular links between Nit1 and these molecules need to be further studied to fully understand the function of Nit1 in NSCLC.

## Figures and Tables

**Figure 4:**
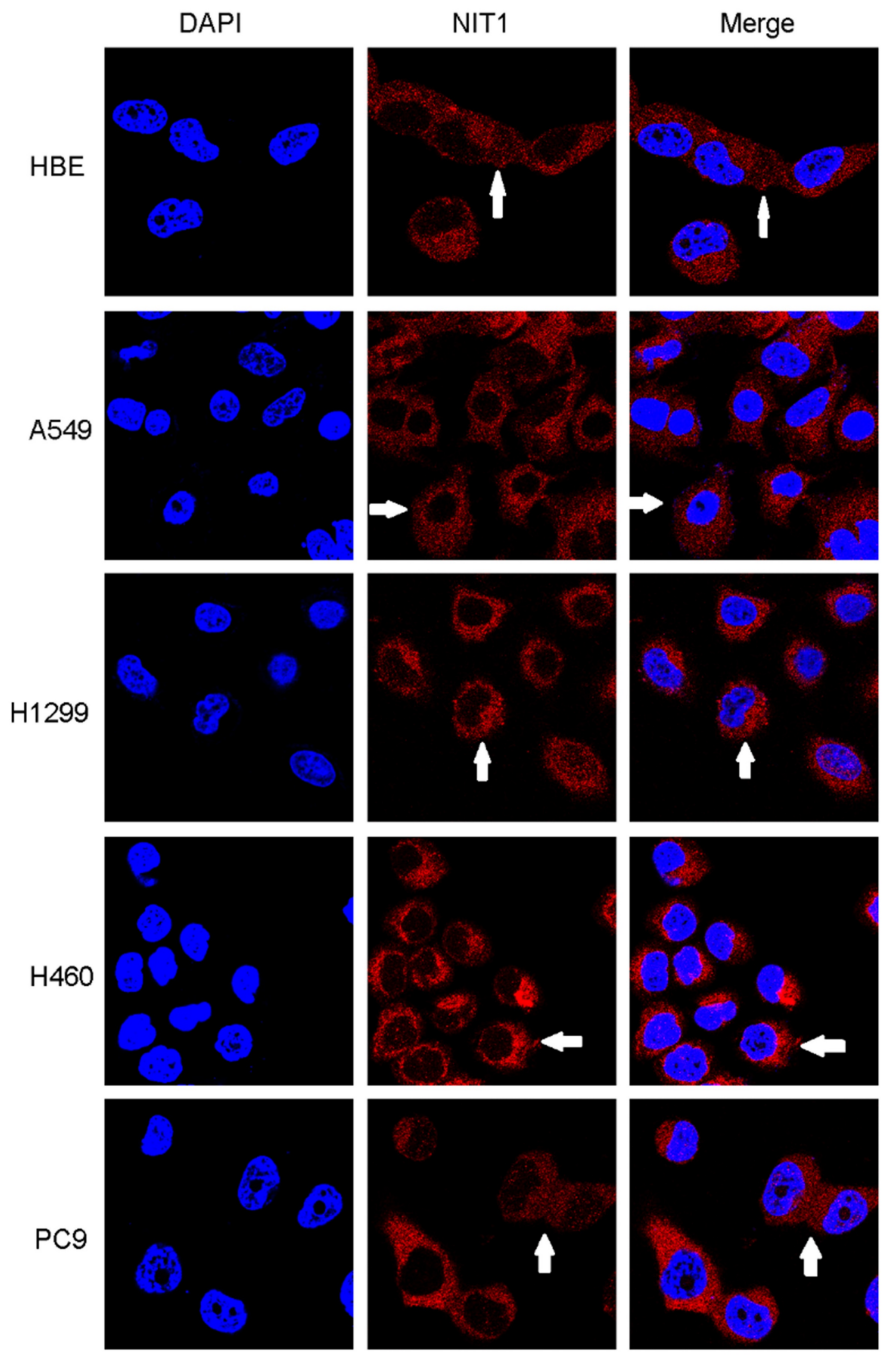
**Detection of NIT1 expression in cell lines *in vitro* using immunofluorescent staining.** NIT1 expression in HBE, A549, H1299, H460 and PC9 cells. The white arrows show that NIT1 was located in the cytoplasm of the cells (×400)

**Table 1:** NIT1 expression in NSCLC and association with clinicopathological features

Characteristics	Numbers	NIT1 Negative	NIT1 Positive	*p*
**Total**	93	33 (35.5%)	60 (64.5%)	
**Age (years)**				
<60	22	8 (36.4%)	14 (63.6%)	0.921
≥60	71	25 (35.2%)	46 (64.8%)	
**Gender**				
Male	55	17 (30.9%)	38 (69.1%)	0.267
Female	38	16 (42.1%)	22 (57.9%)	
**Histological type**				
Squamous cell carcinoma	41	14 (34.1%)	27 (65.9%)	0.811
Adenocarcinoma	52	19 (36.5%)	33 (63.5%)	
**Differentiation**				
Well	19	11 (57.9%)	8 (42.1%)	0.022
Moderate and poor	74	22 (29.7%)	52 (70.3%)	
**TNM stage**				
I and II	52	25 (48.1%)	27 (51.9%)	0.004
III	41	8 (19.5%)	33 (80.5%)	
**Lymph node status**				
Positive	41	9 (22.0%)	32 (78.0%)	0.015
Negative	52	24 (46.2%)	28 (53.8%)	

## Abbreviations

EMT: Epithelial-Mesenchymal Transition
Nit1: Nitrilase homolog 1
NSCLC: non-small cell lung cancer
WHO: World Health Organization
